# The Effect of Facilitated Tucking during Endotracheal Suctioning on Procedural Pain in Preterm Neonates: A Randomized Controlled Crossover Study

**DOI:** 10.5539/gjhs.v6n4p278

**Published:** 2014-05-04

**Authors:** Mona Alinejad-Naeini, Parisa Mohagheghi, Hamid Peyrovi, Abbas Mehran

**Affiliations:** 1 Department of Neonatal Intensive Care Nursing, School of Nursing and Midwifery, Tehran University of Medical Sciences, Tehran, Iran; 2 Department of Pediatrics, Division of Neonatology, Newborn Intensive Care Unit (NICU), Hazrat Rasoul Akram Hospital, Iran University of Medical Sciences, Tehran, Iran; 3 Center for Nursing Care Research, Department of Critical Care Nursing, School of Nursing and Midwifery, Iran University of Medical Sciences, Tehran, Iran; 4 School of Nursing and Midwifery, Tehran University of Medical Sciences, Tehran, Iran

**Keywords:** endotracheal suction, facilitated tucking, preterm neonate, procedural pain

## Abstract

**Background:**

Premature infants not only feel and understand the pain, but also respond more intensively compared with term infants. Non-pharmacological methods of pain control are suitable to relieve pain in painful procedures. The facilitated tucking position is considered as a non-pharmacological method of pain control in infants; however, its impact on frequent and repeated procedural pain such as endotracheal suctioning remains to be studied.

**Objectives:**

This paper is the report of a study that examined the impact of facilitated tucking position on behavioral pain during suctioning in premature neonates. Design: This was a clinical trial study with a crossover design. Settings: The study was conducted in a level II Neonatal Intensive Care Unit, located in a teaching hospital, affiliated to Tehran University of Medical Sciences, Tehran, Iran. Participants: Thirty four infants were enrolled in this study based on the following inclusion criteria: age between 29 to 37 weeks of gestational age, birth weight 1200 grams or more, having an endotracheal tube, no congenital anomalies, no seizures diagnosis, no chest tubes, no intracranial hemorrhage higher than degree II, not receiving opiates and sedatives four hours before intervention and not receiving any painful procedure at least half an hour before the intervention. Methods: The samples were randomly received a sequence of suctioning with/without or suctioning without/with facilitated tucking. Preterm Infant Pain Profile (PIPP) was used to collect the data. SPSS version 16.0 for Windows (SPSS Inc., Chicago, IL, USA) was used for statistical analysis.

**Results:**

While 38.2% of infants experienced severe pain during suctioning without intervention, only 8.8% of them experienced severe pain during suctioning with intervention. The results of the paired t-test show that there is a statistically significant difference in the mean scores of pain between non-intervention and intervention cases (p<0.001), and the mean pain score substantially reduced in cases with intervention.

**Conclusions:**

Given the multiplicity of endotracheal suctioning frequency and the impossibility of frequent use of pharmacological methods of pain relief, the facilitated tucking position can be used as a safe non-pharmacological method for procedural pain management.

## 1. Introduction

Annually, about 13 million premature infants are born worldwide which represents 9% of total births (Als, 2009). This rate reaches to 40% in underdeveloped and developing countries. Virtually All newborns less than 27 weeks old, 80% of infants of 27 to 30 weeks old and approximately 30% of infants between 30 to 32 weeks old need endotracheal intubation immediately after birth (Muller et al., 2003). It is known that respiratory support and related procedures, such as endotracheal suctioning in children provoke response to stress by the endocrine glands. Research has shown that premature neonates not only feel and understand the pain, but also respond more intensively compared with term neonates (Hill & Latour, 2005; Menon & McIntosh, 2008).

During hospitalization in Neonatal Intensive Care Unit, premature neonates require more frequent routine care and procedures than term neonates, therefore, the hypersensitivity elongates and the pain threshold reduces. As a result, non-noxious stimuli such as changing the position and performing routine care may become painful for them and causes stress responses (Hill & Latour, 2005). Inadequate pain management in infants may lead to permanent changes in the process of organizing the brain and appearing maladaptive behaviors (Anand, 2000). Pain may also have detrimental effects on child’s future abilities to learn and remember new information (Weinstein, 2001).

Nurses and other health care team members need to know how to control pain with pharmacological and non-pharmacological approaches (Hill & Latour, 2005). The American Academy of Pediatrics recommends (2000) to use painful procedures in a neonatal intensive care unit only when necessary.

Pain in neonates is controlled by pharmacological and non-pharmacological methods. To control severe pain, some medications such as opiates may be used; however, the use of drugs is not without risk and may cause symptoms such as respiratory depression, nausea, seizures and physiological dependence (Schellack, 2011). These side effects may justify the inadequate management of pain in preterm infants (Hill & Latour, 2005). Non-pharmacological methods of pain control are suitable to relieve pain in painful procedures, since they have short-term impact and are well-tolerated (Menon & Mcintosh, 2008).

Reducing light and noise and changing positions as a part of the developmental care program, aimed at increasing the infant’s energy to cope with painful procedures, are recommended as one of the pain management strategies (Axelin, 2010). Providing direct support like touching the infant helps to the developmental behaviors, and the neonate can better adapt to the life stress out of the womb (Axelin, Salantera, & Lehtonen, 2006). According to Cignacco et al. (2012), the use of touch and positioning the infant is among these supports, which is called facilitated tucking position. Recent studies have shown that the facilitated tucking position reduces pain during blood sampling in neonates (Corff, Seideman, Venkataraman, Lutes & Yates, 1995). The facilitated tucking position includes keeping newborn with warm hands as a tactile and thermal stimulus, which reduces pain during the aggressive procedures (Liaw et al., 2012). Although there are studies that support the effectiveness of developmental care, but there are few studies on confirming the impact of these procedures in frequent and repeated pains (Cignacco et al., 2007).

## 2. The Study

### 2.1 Aim

The aim of this study was to evaluate the impact of facilitated tucking position on behavioral pain during suctioning in premature neonates admitted to the neonatal intensive care unit. In this study, the following hypothesis was tested:

“The score of pain during suctioning in facilitated tucking position is less than the pain score during suctioning alone.”

### 2.2 Design

This was a clinical trial study with a crossover design, in which subjects randomly received a sequence of either suctioning with intervention- suctioning without intervention or suctioning without intervention- suctioning with intervention.

### 2.3 Participants

The study was conducted in a level II Neonatal Intensive Care Unit, affiliated with Tehran University of Medical Sciences, Tehran, Iran. Sampling was done purposefully by the main researcher from January 2013 to May 2013, based on the following inclusion criteria: age between 29 to 37 weeks of gestational age, birth weight 1200 grams or more, having an endotracheal tube, no congenital anomalies, no seizures diagnosis, no chest tubes, no intracranial hemorrhage higher than degree II, not receiving opiates and sedatives four hours before intervention and not receiving any painful procedure at least half an hour before the intervention. Thirty four neonates who met the inclusion criteria were enrolled in this study. After that, random sequencing was done to determine the intervention- non intervention receiving order for each neonate by the main researcher.

### 2.4 Data Collection

Demographic characteristics form and Preterm Infant Pain Profile (PIPP) were used to collect the data. The information on gestational age, weight, underlying diseases and the Apgar Score at fifth minute and the method of delivery were collected using the demographic characteristics form. Given that the dependent variable in this study was pain during suctioning, the PIPP tool was used to measure this variable. The PIPP is a relatively easier tool which does not require extensive training (Hill et al., 2005; Ballantyne, Stevens, McAlister, Dionne; & Jack, 1999). The PIPP is used to assess procedural pain and includes heart rate, oxygen saturation, closing the eyes, nose-lip chin, brow bulge as well as gestational age and the mode of behavior for pain assessment (Obeidat, Kahalaf, Callister, & Froelicher, 2009). Considering that arterial oxygen saturation and heart rate are used to determine the pain score, these values were observed through the monitor and pulse oximeter attached to the patient and recorded on the pain scores form. Scores given to each of the items can be from zero to three, and the pain total score can range from zero to 21. Zero to six scores indicate a lack of pain, seven to twelve score indicate mild pain and scores higher than 12 indicate of moderate to severe pain (Verklan & Walden, 2010; Ballantyne et al., 1999). Time needed for observing the infant regarding the mode of behavior was 15 seconds, and behavioral and physiological criteria needed 30 seconds each. The instrument has a high reliability and validity (Hill et al., 2005).

### 2.5 Intervention

The university ethics committee approved the study. The researcher provided a full explanation about the study and its purposes for the head nurse and personnel as the researcher assistants. Considering that implementing the intervention and variables measurement needed another individual presence with the principal researcher, the researcher assistants help the main researcher at all stages of performing intervention and the measurements.

After selecting the subjects, written informed consent was taken from the parents of the neonates, and demographic data were collected and recorded from their medical records. Half of the neonates under study initially received suctioning without intervention and then, suctioning associated with intervention, and the other half initially received suctioning with intervention and then, suctioning with no intervention. Given that suctioning was done based on the infant’s need, just in such cases, the researcher measured data, performed suctioning and intervened.

**Figure 1:**
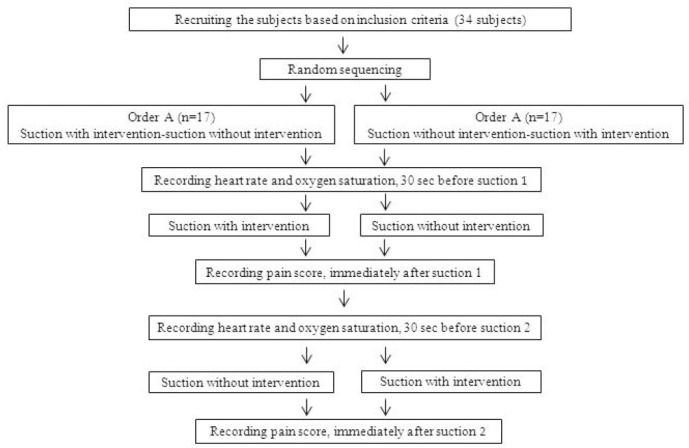
The steps of the study

The neonates were kept in a quiet environment during the intervention, and it was tried to minimize the environmental stimuli such as light and sound at all stages of the study and keep them alike. In cases the neonates recruited for the study need suctioning, the researcher first was setting the video camera to cover the infant’s whole body in its filming filed while the face was toward the camera. Then, the recently calibrated pulse oximetry probe was attached to the infant’s right foot followed by recording the heart rate and oxygen saturation from the monitors, 15 seconds before the start of suctioning (as baseline data to calculate pain scores immediately after suctioning). Scores given to the infant’s facial expression immediately after suctioning, together with heart rate and oxygen saturation levels were used to calculate the PIPP score. After filming, the recording of each neonate was watched and coded by the first researcher. Cases with and without intervention were separated and scored by the second researcher in two consecutive days. The steps of the study are shown in Figure 1.

In cases when the neonates were prescribed to receive suctioning without intervention, the researcher assistant performed the suction and the researcher only recorded the study variables. In cases where the neonates were receiving suctioning with intervention, the suctioning was carried out by the researcher assistant and the researcher implemented the intervention (placing the neonate in facilitated tucking position). To implement facilitated tucking, the neonate was placed on his/her side, his/her back was gently bent, the legs were getting up and flexion at an angle greater than 90 degrees, the shoulders were also constricted up to 90 degrees and the researcher hands were placed over the head close to the mouth or on the neonate’s face. The interval between suctioning without intervention and suctioning with the intervention was at least two hours.

### 2.6 Data Analysis

SPSS version 16.0 for Windows (SPSS Inc., Chicago, IL, USA) was used as a platform for statistical analysis. Descriptive statistics were used to present a general picture of participants’ demographics. Means (with/without intervention) were compared using a paired t - test.

## 3. Results

Demographic characteristics of neonates are shown in Table 1. Most of the neonates were male (52.9%), 1 to 7 days old (79.4%), born by caesarean (73.5%), with gestational age less than 32 weeks (59.9%) and weight less than 1500 grams (73.5%), and also, with fifth minute Apgar score 8 to 10 (52.9%).

**Table 1:** Demographic characteristics (infants served as their controls)

Variable	Frequency (percent)
**Gender**	
Male	18(52.9)
Female	16(47.1)
**Postnatal age (day)**	
1-7	27(79.4)
8-14	2(5.9)
≥15	5(14.7)
**Type of delivery**	
Normal vaginal delivery	9(26.5)
Cesarean	25(73.5)
**Gestational age (week)**	
<32	19(55.9)
32-36	7(20.6)
>36	8(23.5)
**Weight (gram)**	
<1500	25(73.5)
1500-2500	6(17.6)
>2500	3(8.8)
**5-minute Apgar score**	
4-5	7(20.6)
6-7	9(26.5)
8-10	18(52.9)

Table 2 shows the distribution of pain levels in patients received suction with and without facilitated tucking position. While 38.2% of neonates experienced moderate to severe pain during suctioning without intervention, only 8.8% of them experienced moderate to severe pain during suctioning with intervention.

**Table 2:** Pain level frequencies during suctioning without/with facilitated tucking

Pain level	Nonintervention N (%)	Intervention N (%)
No pain	0(0)	7(20.6)
Mild	21(61.8)	24(70.6)
Moderate to Severe	13(38.2)	3(8.8)

The results of the paired t-test show that there is a statistically significant difference between the mean scores of pain in non-intervention and intervention cases (p< 0.001), and the mean pain score substantially reduced in cases with intervention (Table 3).

**Table 3:** Comparison of PIPP score during suctioning without/with facilitated tucking

Variable	Nonintervention		Intervention		t value	P value

	Mean	SD	Mean	SD		
PIPP score	11.88	3.05	9.06	2.95	4.41	0.001

## 4. Discussion

One of the limitations of this study was to include only the infants with gestational age 29 weeks and older, and we do not know whether the facilitated tucking position is effective for younger or more severely ill babies or not. Although data collection was done by one researcher and scoring was done by another researcher and the second researcher was not aware of the scores in cases with and without intervention, but the researcher responsible for the pain scoring was aware of the study objectives.

Intensive care for neonates includes performing of many diagnostic and therapeutic procedures, which are associated with pain. Thus, prevention and treatment of neonatal pain are of great importance in the neonatal intensive care unit (Gitto et al., 2012). The premature neonates have a little ability to put themselves in a flexion position during stressful events, and thus, they cannot use this self-comforting strategy to cope with their stress. The facilitated tucking position allows the premature neonate to keep his/her motion, physiological and autonomic stability much more better (Hill et al., 2005).

In this study, the pain during endotracheal suctioning was taken into account, for this painful procedure is commonly carried out in the neonatal intensive care unit, but it has been neglected in previous studies (Axelin et al., 2006). Results from this study showed that the pain due to suctioning procedure is considerably reduced by applying the facilitated tucking position. In a study in 2005 by Axelin and Salantrera to examine the effect of the facilitated tucking position by parents on premature infants’ pain during suctioning, it was found that this intervention decrease the pain during the endotracheal suctioning procedure. In this study, the Neonatal Infant Pain Scale (NIPS) tool was used, which provided results similar to the finding of the present study using the Premature Infant Pain Profile (PIPP) tool. A study was conducted by Ward-Larson, Horn and Gosnell (2004) to examine the effect of the facilitated tucking position on behavioral pain of neonates with very low birth weight during routine care measures. The pain measuring tool in this study was PIPP, and the study found that the facilitated tucking position has reduced the pain scores.

Based on the results of the study regarding pain levels, it was found that in cases of using facilitated tucking position, the frequency of neonates with severe pain has dropped, and the neonates have experienced mostly mild to moderate pain with this position, while in cases of doing endotracheal suctioning without intervention, they had experienced mostly moderate to severe pain.

Given the multiplicity of endotracheal suctioning frequency and the impossibility of frequent use of pharmacological methods of pain relief, the facilitated tucking position can be used to reduce pain during the suctioning procedure in premature neonates. The presence of pain assessment tools such as the PIPP, which can be easily used by nurses, will help them to document the impact of their pain management action on neonatal pain.

## 5. Conclusion

The best method of pain management in infants includes the prevention of pain with clinical judgment about the need to do any painful procedure, clustering the procedures and performing each procedure slowly. In addition, every painful procedure should be combined and associated with an effective and safe pain relieving approach. Findings from this study show that the facilitated tucking position can be used as a non-pharmacological and safe method of procedural pain management.
